# Carbon Sequestration to Avoid Soil Degradation: A Review on the Role of Conservation Tillage

**DOI:** 10.3390/plants10102001

**Published:** 2021-09-24

**Authors:** Sadam Hussain, Saddam Hussain, Ru Guo, Muhammad Sarwar, Xiaolong Ren, Djordje Krstic, Zubair Aslam, Usman Zulifqar, Abdur Rauf, Christophe Hano, Mohamed A. El-Esawi

**Affiliations:** 1 Department of Agronomy, University of Agriculture Faisalabad, Faisalabad 38040, Pakistan; ch.sadam423@gmail.com (S.H.); sarwar1406@gmail.com (M.S.); zauaf@hotmail.com (Z.A.); usmanzulfiqar2664@gmail.com (U.Z.); 2 College of Agronomy, Northwest A & F University, Yangling, Xianyang 712100, China; guoru@nwafu.edu.cn (R.G.); rxlcxl@aliyun.com (X.R.); 3 Faculty of Agriculture, University of Novi Sad, 21000 Novi Sad, Serbia; djordje.krstic@polj.uns.ac.rs; 4 Department of Chemistry, University of Swabi, Anbar 23430, Pakistan; mashaljcs@yahoo.com; 5 Laboratoire de Biologie des Ligneux et des Grandes Cultures (LBLGC), INRAE USC1328, Université d’Orléans, 28000 Chartres, France; hano@univ-orleans.fr; 6 Botany Department, Faculty of Science, Tanta University, Tanta 31527, Egypt

**Keywords:** carbon sequestration, climate change, global warming, soil conservation, zero tillage

## Abstract

Human efforts to produce more food for increasing populations leave marks on the environment. The use of conventional agricultural practices, including intensive tillage based on the removal of crop residue, has magnified soil erosion and soil degradation. In recent years, the progressive increase in the concentration of greenhouse gases (GHGs) has created global interest in identifying different sustainable strategies in order to reduce their concentration in the atmosphere. Carbon stored in soil is 2–4 times higher than that stored in the atmosphere and four times more when compared to carbon stored in the vegetation. The process of carbon sequestration (CS) involves transferring CO_2_ from the atmosphere into the soil or storage of other forms of carbon to either defer or mitigate global warming and avoid dangerous climate change. The present review discusses the potential of soils in sequestering carbon and mitigating the accelerated greenhouse effects by adopting different agricultural management practices. A significant amount of soil organic carbon (SOC) could be sequestered by conversion of conventional tillage to conservation tillage. The most important aspect of conservation agriculture is thought to improve plant growth and soil health without damaging the environment. In the processes of climate change mitigation and adaptation, zero tillage has been found to be the most eco-friendly method among different tillage techniques. No-till practice is considered to enable sustainable cropping intensification to meet future agricultural demands. Although no-tillage suggests merely the absence of tillage, in reality, several components need to be applied to a conservation agriculture system to guarantee higher or equal yields and better environmental performance than conventional tillage systems.

## 1. Introduction

Global warming driven by anthropogenic emissions of greenhouse gases (GHGs) has increased Earth’s temperature and is likely to exceed 1.5 °C by the end of this century [1,2]. Due to global warming effect, large-scale shifts in weather patterns may affect terrestrial C storage. The net primary production C inputs to the soils and soil C decomposition rates are significantly affected by the change in temperature, precipitation, and CO_2_ concentration. Changing climatic patterns may also accelerate land-use change and, thus, alters the terrestrial C fluxes [3]. A growing body of evidence indicates that agriculture production has grown on average between 2 and 4% annually in the past 50 years, while the area under crop cultivation has increased by only 1% per year with a decline of 0.44 ha to <0.25 ha per capita arable land globally [4], which indicates successful agricultural intensification. Continuous monoculture and extensive farming practices, unavailability of good quality seed, inappropriate crop rotation, and various environmental factors have resulted in the degradation of soils and other natural resources, which affect global food security and livelihood opportunity available to small-scale family farmers [5]. Traditional tillage practices, including deep ploughing, are highly destructive for agricultural soils and result in 24% of global land degradation [6]. Agricultural soils are particularly more vulnerable to erosion because of the removal of most of the vegetation in a conventional tillage system. In this context, Lal [7] studied the yield levels under different erosion conditions and reported that accelerated soil erosion has reduced the yield by 29% over 25 years (1995–2020) under rainfed agriculture. According to an estimate, about 30% of arable land is severely degraded by soil- and wind erosion [8], and 10 million ha of arable land is lost due to accelerated soil erosion each year on the globe [9]. Furthermore, it is reported that soil degradation resulted in 16–20% C loss of the present-day global soil C stock (1200–1500 Pg) to a one-meter soil profile over 1000 years in the past [10]. According to Lal [11], intensive and continuous tillage practices resulted in soil C loss, and 60–90 Pg of soil organic C (SOC) was globally lost during the past several decades. In addition, Lal [12] demonstrated that the excessive use of farm machinery has resulted in the depletion of soil C pool by 66–90 Pg of C since 1750 and deforestation has depleted soil C stock by 22%. The decompositions of biomass and soil C stock are the key sources of CO_2_ emissions during the past few decades [13]. Therefore, in order to reduce the problems associated with land degradation, adopting sustainable approaches is inevitable in order to secure natural resources and ensure food security without affecting the environment.

Carbon sequestration (CS) is a method of reducing the carbon dioxide (CO_2_) emissions into the atmosphere by involving long-term storage of CO_2_ in soil, which helps to mitigate the global warming effects [14,15]. Carbon sequestration is associated with transferring atmospheric CO_2_ into the soil in the form of stable C pools [16,17,18]. The sequestration process mainly takes place through the humification of biosolids and crop residues added to the soil and the formation of secondary carbonates by the dissolution of CO_2_ [19]. In the same study, authors further reported that leaching of bicarbonates into groundwater also contributes to reducing the emissions of captured CO_2_ [19]. In a terrestrial environment, carbon sequestration is defined as the net removal of atmospheric CO_2_ and its storage as stable C pools in the soil. According to Kumar et al. [14], the stable C pools contain soil living biomass, including roots, above-ground plant biomass, and recalcitrant inorganic and organic C compounds in soils and deeper subsurface surroundings.

Soil C sequestration is also necessary for improving soil nutrition status and increasing resource use efficiency of crop plants to ensure better growth and productivity in a sustainable manner. Through denaturation and filtration of pollutants, soil C sequestration also helps to improve soil water status and enhances biodiversity by protecting land for nature conservancy. However, a lack of an accurate estimation of soil C sequestration and inherent soil variability, including special heterogeneity of SOC, hinder the identification of suitable management practices [20]. In terrestrial ecosystem, different studies have determined that the main factors influencing the spatial variability of SOC were soil types, climate, topography, land use patterns, tillage practices, and fertilizer application [21]. The availability of inorganic colloid in the soil is the core substrate to sequester C [22]. The adaptation of sustainable management practices, including a suitable crop system, proper nutrient management, and adapting conservation tillage practices, can help to ameliorate the constraints associated with soil C sequestration [23,24]. The maintenance of soil fertility in a sustainable manner is also essential for improving the soil structure, enhancing soil productivity and soil organic C content. The higher C content can determine soil quality and has significant influence on soil physio-chemical and biological properties. Therefore, it is necessary to maintain and restore the soil’s C stock mainly through conservation tillage practices, which can be helpful in mitigating the adverse effects of climate change and reduce degradation problems.

In recent years, conservation agriculture (CA) is considered as an alternative to conventional agriculture in many parts of the world. It is reported as an efficient method for improving crop performance and soil properties and induces positive effects on climate change mitigation [25,26]. Conservation agriculture, including the use of woody crops and residue-based zero tillage, improves the water infiltration into deeper soil layers, moderates soil temperature, prevents soil erosion, reduces weed infestations, improves soil aggregation, minimizes soil compaction, increases soil organic matter contents, reduces the emission of GHGs, decreases the production costs, and maintains some fallow through direct seeding [27,28]. This practice protects natural resources and sustains crop productivity, and long-term use of residue-based zero tillage is an effective approach for sequestering environmental CO_2_ into the soil, which maintains high crop yields [24,29,30].

Zero tillage (ZT) with surface maintenance of crop residues is also reported to be effective in minimizing soil disintegration, in enhancing soil fertility status and the SOC sequestration potentials in adapting, and mitigating global warming’s effect [24,31]. However, its effectiveness depends highly on the quantity and quality of crop residue mulch returned to the field [32]. In a no-till farming system where the biomass production numbers less than the output, the rate of SOC sequestration is negative. The SOC sequestration rates for no-till farming in diverse ecosystems vary widely among ecosystems and range from 0 to 1000 kg ha^−1^ yr^−1^ [12,32,33,34,35,36]. Based on the average data of 67 long-term experiments, West and Post [36] reported that no-till farming systems sequester the carbon with an average rate of 57 ± 14 g C m^−2^ yr^−1^. However, de Torres et al. [37] reported that SOC increased by cover crops between 10.9 and 14.3 Mg ha^−1^ in four seasons. Some studies also reported that rates of SOC sequestration vary with soil layers, with peak sequestration rates in the soil surface layer [13], up to 40 cm soil profile [38]. However, the data need to be carefully re-examined in order to exceed the effect of no-till system on the distribution of soil C among different soil layers [39]. In addition to soil depth, soil properties (including clay content and type, fertility status, and soil water retention); landscape orientation (less in shoulder and summit slopes than foot slopes); profile and terrain characteristics (more in young soils with deep effective rooting depth and less in south-facing and convex slopes than in north-facing and concave slopes); and climatic conditions (for temperature, less in warm than cold climates and, for rainfall, less in dry than humid regions) also determine the soil CS rates. The objective of the manuscript is to review the potential of soils in sequestering C and mitigating the accelerated greenhouse effects by adopting conservation agriculture practices. A major focus was given on the extent and scope of SOC sequestration by shifting from conventional tillage to conservation tillage.

## 2. Major Causes and Factors for the Depletion of Soil Organic Carbon

The conversion of natural to agricultural ecosystems is rapidly depleting the SOC pool. The magnitude of SOC pool depletion is 25–50% over 20 to 50 years in soils of the temperate climatic zones, and 50–75% over 5 to 20 years after deforestation in soils of the tropic climates [40]. The magnitude of depletion is low under the condition where the C inputs in managed ecosystems (through crop residue retention, along with the application of other biosolids) are higher than the outputs. The latter comprises losses of SOC through accelerated erosion (results from human-induced removal of natural vegetation), mineralization, and leaching. Agricultural soil erosion has been reported to agitate global carbon cycle through loss of SOC by transporting organic carbon-rich sediment off an agricultural land unit and surface runoff [41]. The rate and magnitude of SOC depletion are tremendous for structurally inert soils. Among these, Kaolinite soils are reported to deplete SOC more rapidly because these soils comprise low content of low activity clays, poor fertility status and water holding capacities, uncontrolled internal drainage, poor soil structure along with weak and low aggregation, and overall low agronomic/biomass productivity. Furthermore, landscape position also has a significant impact on the SOC pool and its dynamics through avoiding water runoff and soil erosion. Along with physiographic and pedologic components, numerous socioeconomic and political factors, including lack of visionary and committed leadership, poor policy incentives, and no extension support, also play a significant role in the rate and magnitude of SOC depletion [42]. Lower SOC depletion is reported under agricultural systems in which balanced input/output ratios are exits. A subsistence farming system, with fertility-mining and extractive agricultural practices, in which the output exceeds the input can cause relatively higher SOC depletion than the system with balanced input/outputs ratios. The magnitude of SOC depletion also varies among tillage practices, and relatively low depletion is reported for no-till mulch farming system and the application of organic fertilizers than compared to plow-based tillage system with lack of straw retention [19]. Moreover, a positive correlation between the depletion of the SOC pool and the emission of CO_2_ has been reported [43]. The depletion of the SOC pool is reported to have a negative impact on soil quality and the balance between the elemental and nutrient. It also disturbs soil water balance through runoff losses and high evaporation rates, and it can induce severe reduction in soil biodiversity, including the activity of soil microorganisms. According to Lal [44], the decline in soil quality negatively influences the net primary productivity and lowers the quality and quantity of produced plant biomass to cause a severe depletion of the SOC pool.

## 3. Management of Soil Organic Carbon

The term SOC sequestration is defined as the process of transferring atmospheric CO_2_ into the soil C pool through humification of crop residues and other soil organic materials (e.g., biosolids), which are not immediately re-emitted back into the air [17]. SOC sequestration could be achieved by the following: (i) retaining crop residue (below and above-ground plant biomass) within the soil to be converted into organic carbon; (ii) increasing crop growth for more residue retention; (iii) reducing decomposition and soil erosion to protect and stabilize organic carbon content; and (iv) enhancing soil C budget by increasing synergisms between crop plants, soil, and atmospheric processes in order to gain saturated soil C sink capacity. Increasing SOC content and its management through soil-based and crop-based management practiced by the application of C-enriched material (including biochar and mulches) and organic fertilizers and judicious use of land resources are key factors that determine the SOC sequestration [45,46].

Low carbon agriculture practice (LCA) is referred as a sustainable approach for protecting the environment, improving agronomic crop yields, and minimizing global warming effect. The progressive increase in soil organic matter content increases the availability of the main nutrients for better growth and productivity. Low C agricultural practices are characterized by reduced emissions of GHGs (including CO_2_) and high storage of SOC and vegetation. The strategy refers to the adoption of best management practices that protect the environment and natural resources and ultimately crops yield. It is among the most suitable approaches for mitigating GHG emissions [47,48,49]. Low C agriculture practice is primarily based on the adaption of the best agricultural management practices to reduce the CO_2_ emissions from land use. In addition, LSA also reflects the efficient use of energy resources by involving following operations: (i) decreasing the fossil fuel input by adaption of no or reduced tillage practices, (ii) enhancing nutrient use efficiency by increasing crop diversity and use of cover crops, and (iii) strengthening biological N fixation by including legume crops in crop rotation [50,51]. It has been reported as a sustainable strategy based on decreasing use of chemical N fertilizer [51], expansion of the area under reduced or no-tillage, and the use of crops with high biomass-C input [47,49].

The leakage of C sequestered in the soil is often discussed in terms similar to the sequestration of CO_2_ injected in geological strata. SOC sequestration is a natural process and is subjected to leakage if the recommended land use and soil management practices are discontinued. Long-term adaptation of sustainable land use and soil management practices significantly enhances the residence time of SOC. The use of minimum or reduced tillage practices provides continuous soil cover; incorporation of crop residues and application of biosolids are among the essential factors for enhancing residence time of SOC [19]. Residence time can also be enhanced through the development of deep root system owing to the incorporation of SOC in subsoil, which creates positive soil nutrient balance and improves soil health. According to Lal [19], there are three basic principles for achieving higher residence time through conversion of marginal or degraded lands to perennial vegetation. The conversion of traditional tillage to conservation tillage can increase the efficiency of agricultural ecosystems.

## 4. Mechanism of Soil C Sequestration

The process of soil carbon sequestration involves three basic mechanisms including the formation of soil micro-aggregates, its long-term stability, and improvement in soil structure with the deep placement of SOC in the sub-soil layers [52,53,54,55]. These processes are commonly addressed as physical and chemical mechanisms. The formation of clay domains and micro-aggregates and cementation of primary particles is based on the foundation of organo-mineral complexes. According to Lal and Kimble [52], these organo-mineral complexes bind clay into aggregates and after immobilizing helps in carbon sequestration. Micro-aggregate dynamics are influenced by the humic substances and other persistent compounds, including polymers [56,57,58]. A stabilization of macro-aggregates can protect soil organic matter (OM) against soil microbial activity. Clay content and mineralogy have long-lasting effects on aggregation. Additionally, Beare et al. [59,60] reported a positive correlation between total SOC concentration and aggregate size. Furthermore, climatic conditions, soil properties, tillage practices, and availability of soil nutrients also define the humification efficiency of biomass C. Humification efficiency of biomass C is less in warm and dry climates than in cool and humid regions. Additionally, coarse-textured soils with a low surface area have lower humification efficiency than clayey soils with a high surface area. No-till farming system positively influenced the humification efficiency. Working with maize crops in Coshocton, Ohio, Puget et al. [61] reported that the fraction of total C in crop residue converted to SOC was 11.9% for no-till practice and 8.3% for plow tillage crops. Similarly, in another study, Allmaras et al. [62] studied that humification efficiency for no-till soils was 26% compared with 11% for traditional tillage practices, including chisel and moldboard plow application. The availability of soil nutrients, such as nitrogen (N), phosphorus (P), sulphur (S), zinc, and copper, also affects the humification efficiency because C is the main building block of humus. Himes [63] reported that ~28 Mg of C in 62 Mg of oven-dry residue is needed to sequester the 10 Mg of C in crop residue into 17.241 Mg of humus. Furthermore, the authors further added that it requires 833 Kg N, 200 Kg P, and 143 Kg S. Thus, the availability of essential nutrients, including N, P, and S, is mandatory for the humification of residue C. In this context, Jacinthe et al. [64] reported that residue-C conversion into SOC was 32% with fertilizer application compared with 14% without fertilizer application for Luvisol in central Ohio. Under the soils with mulch application, a similar amount of SOC stocks (25.6 Mg C ha^−1^) has been recorded both for with and without application of fertilizers. However, with mulch application, additional accretion of SOC occurs only where additional fertilizer was applied. The adoption of the no-till system does not essentially increase the SOC pool without adequate fertilization [65]. The application rates and placement of N fertilizer have a significant influence on the SOC sequestration rate [66,67,68]. The illuviation as well as translocation of C into sub-soil layers is another important mechanism. Bioturbation by earthworm, termites, and the deep root system results in deep translocation of C away from the anthropogenic zone and climatic disturbances [69,70]. The activities of soil invertebrates along with aggregate formation and soil clay-C interactions determine the formation and characteristics of biogenic structures that later degrade due to climatic factors. Continuous tillage practices can result in reducing the mean weight diameter of soil aggregates to facilitate the erosion process. The dissolved organic carbon has numerous sources from below ground and above ground and flows through the land to the aquatic streams.

## 5. Conventional Tillage and Soil Carbon Stocks (CS)

**Figure 1:**
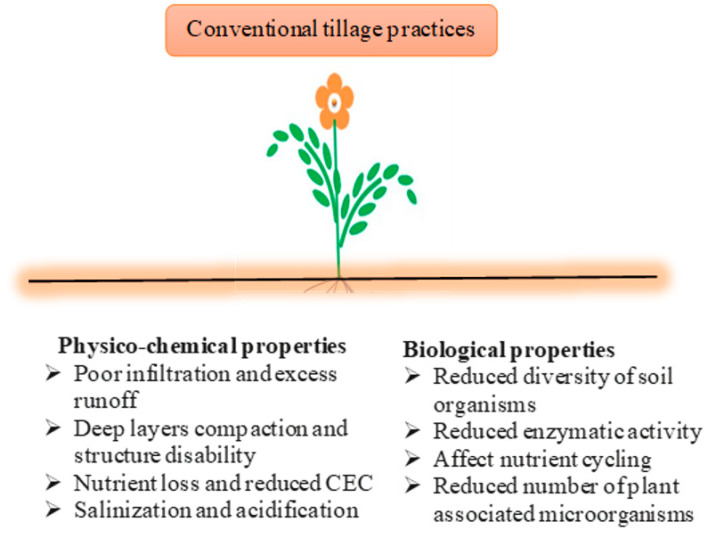
The impact of conventional tillage practices on the soil environment. Note: CEC denotes cation exchange capacity.

The main objective of any tillage practice is to supply a favorable soil environment for better plant growth and development. Tillage is one of the major factors that determine soil C stocks. The intensive tillage practices result in significant reduction in SOM (Figure 1). According to Kumar et al. [14], the SOM is oxidized and results in reducing the soil organic matter (OM) content under intense mechanical cultivation unless supplementary OM is added to the soil. Oxygen is introduced when soils are exposed to air by tillage, which stimulates SOM decomposition by soil microbes, while removal of ground cover exposes the organic-rich topsoil layers to wind and water erosions [71]. Intensive tillage practices also disrupt the pores left by plants’ roots and soil microbial activity. The intense mechanical cultivation causes a rapid decomposition process and losses of the SOM, which are protected inside the soil aggregates. Tillage practices also result in the breakdown of the soil aggregates and increase in the oxygen supply and surface area exposure of organic material.

Under conventional agricultural practices, intensive tillage operations have always been reported as a potential contributor in increasing the emission of GHGs, which contributes more to the global warming effect [72]. Intensive tillage practice also loses soil nutrients and organic carbon content and instigates soil disintegration based on the soil surface topography [73,74]. On the globe, the soils contain as much as 2400 Gt C in a depth of 2 m, which is more than three and four times that in the atmosphere and measured C in physical biota, respectively [75]. In an agricultural ecosystem, organic matter is the main reservoir of C. The role of organic matter is also identified as its tendency of ‘C source’ and ‘sink’. Furthermore, it is also reported that organic matter has a positive role in the reservation of soil water. It reduces erosion losses and improves soil biological properties. In agricultural systems, increasing SOC levels helps to decrease CO_2_ emissions and improves soil fertility status and crop productivity; thus, it creates a win-win circumstance [43,76]. Therefore, the conversion of conventional tillage to conservation tillage is a need of the hour in order to reduce the soil disturbance and the emissions of GHGs, including CO_2_ and N_2_O.

Plow tillage results in pulverization and physical disturbance of the soil, which produces fine and loose soil structure compared to reducing tillage that leaves soil intact [77,78,79]. In an agricultural system, tillage is the main source of CO_2_ emission through the biological decomposition of organic matter. It increases the supply of oxygen, breaks soil aggregates, and promotes the decomposition of organic matter by exposing the surface area of organic material. Soil aggregates are closely associated with the physical properties of the soil and SOC sequestration. Soil organic matter is known to improve soil structure and stability by compressing the mineral particles into soil aggregates [53,80]. Soils having good structure are commonly known to have better water-holding capacities, moderate saturated hydraulic conductivity, and adequate aeration for better plant growth and development [81,82]. Furthermore, stable aggregates help in reducing the decomposition of SOM [83,84,85,86].

Soil aggregates fractionation has been extensively recognized to evaluate the stability of organic carbon and the effects of soil management on organic carbon dynamics [87,88]. Agricultural practices, including tillage operations, cropping systems, and fertilizer application, also influence the physical properties of the soil and GHGs emissions [89,90]. Intensive tillage operations reduce aggregate stability, increase soil compaction of deep layers, decrease retention and transmission of soil water and solutes, and aggravate losses due to soil erosion and surface runoff. Intensive tillage practice also results in an increased rate of organic C mineralization to deplete more organic matter, increase erosion losses, and reduce the cycling of organic matter through the removal of ground cover [91,92].

## 6. Conservation Agriculture and NT for Soil Organic Carbon

The conservation agriculture (CA) system has been reported as an alternative method for improving agricultural production in a sustainable manner. This method is widely considered to enhance the infiltration rates, reduce erosion problems, and improve soil quality and organic C contents in agricultural ecosystems [42,93]. In another study, Prasad [94] reported that conservation agriculture also reduces soil degradation problems under rainfed agriculture. Conservation agriculture comprises no-till farming (with minimal soil disturbance) and the use of crop mulches along with appropriate crop rotations [95]. According to FAO, it is also defined as a sustainable approach to enhance resource-saving and agricultural crop production while improving the environment [46]. According to Dumanski et al. [96], conservation agriculture involves a supply of modern agricultural technology to improve crop production and maintain the health and integrity of the ecosystem in contrast to traditional agriculture operations, which mainly aims at maximizing yields habitually at the cost of the environment [96]. The FAO recognized that the CA system enhances sustainable land management, increases crop production without damaging the environment, and mitigates the adverse effects of climate change [97,98]. In recent years, CA has gained more popularity due to its numerous benefits including improved soil physico-chemical and biological properties, better soil fertility and water status, long-term sustainable productivity, and mitigation of climate change [99,100,101]. In upland crop production systems, conservation agricultural practices increase soil water and nutrient status, residual water content, soil infiltration rate, and organic carbon content contrary to traditional systems [102,103]. CA is based on three core principles referred to as no-till practice of minimum soil disturbance, continuous soil cover through mulching, and adaptation of crop rotations and intercropping practices [104]. Some researchers also included the adoptation of woody crops to attain higher yields in low fertility soils without affecting the environment [105]. Lal [106] defined CA on the basis of integrated nutrient management and demonstrated that CA protects the natural eco-system, improves soil nutrient cycling, and reduces the emission of major GHGs, including CO_2_, to reduce the global warming effect. Woody crops have some structural features allowing them to potentially sequester atmospheric C to a significant extent [107,108]. Their long-life cycle allows them to accumulate C in permanent organs such as trunk, branches, and roots and in the soil (e.g., rhizodeposition). In addition, the massive and deep-rooted systems in these perennial woody crops allow direct transfer of SOC into the subsoil, making it less prone to mineralization. Adoption of cover crops (CC) in the cropping system is also recommended as a management strategy for increasing SOC sequestration rate [109,110]. Usually, the increase in organic carbon with cover crop adaptation has been attributed to the increase in organic input through plant residue and to a reduction in mineralization rate due to conservative tillage practices [111,112]. In addition to providing C inputs, cover crops offer numerous agroecosystem services such as reduced N losses via leaching [113], encourage sustainable crop production [114], and improved soil quality [37,115].

Different studies have also reported that CT helps accumulate more soil C in the tropics (torrid zone), temperate, and polar climatic zones [11,36,99]. This method has great potential in enhancing organic carbon and organic matter content in the soil, and it can provide great contributions in increasing SOC sequestration [17,103,116,117,118,119]. According to Mathew et al. [120], CT practices are found to positively influence the physico-chemical as well as the biological properties of the soil. Conservation tillage practices also increase soil respiration rates and microbial biomass production [121]. Das et al. [122] reported that CT significantly reduced soil bulk density and enhanced organic carbon content, microbial biomass C, and sustained the activity of the soil organisms. Mutema et al. [123] also demonstrated that conservation tillage practices with reduced tillage and crop cover enhance macro-fauna activities, their abundance, and diversity. Reduced risk of soil erosion; better soil quality (through enhancing organic matter content); improved fertility status; water infiltration rate and storage capacity; flora and fauna populations; stability of agro-ecosystem; energy use efficiency; and improved crop yield are also reported as a positive influence of CT practices [124,125,126]. Under a no-till system, a substantial amount of C is added to the soil when crop residues are left on the surface of the soil, while minimum tillage reduces soil disturbance, thereby slowing the residues’ incorporation and lowering the susceptibility to physical disruptive forces, which in turn reduce the mineralization rates of organic matter [127]. Similarly, under a reduced tillage system, the retention of crop residues (including stubbles and root biomass) increases SOC, which in turn creates a physical barrier between the substrates and soil microbes to form the stable microaggregates and macroaggregates that protect the microbial decomposition [128].

Under the NT system, mulching also improves soil environment [129,130,131,132] and moderates soil thermal conditions without penalizing crop yield [133,134]. Furthermore, during intense rains, mulching also increases soil porosity and water holding capacity [135,136], reduces the surface runoff, and accelerates soil erosion [137]. In previous studies, it is also well reported that the incorporation of fresh residue promotes the formation of macroaggregates because it provides a good C source for soil microbial populations and the production of microbial-imitative binding agents [83,138]. The essential role of macroaggregates in C sequestration is well reported by Dorodnikov et al. [139]. In general, microaggregates (<0.25 mm) are reported with less C than macroaggregates (>0.25 mm) [140,141,142,143,144]. Adaptation of NT increases the proportion of macro-aggregates (0.25–2 mm) and reduces the proportion of micro-aggregates (0.05–0.25 mm) vis-a′-vis traditional tillage practices. Furthermore, no-tillage practice results in increasing the amount of carbon-enriched macro-aggregates and decreases the amount of carbon-depleted microaggregates [54,59,60,127,140,144]. As compared to plow tillage, the adaptation of NT is also reported to reduce the turnover rate of macro-aggregate, which increases the aggregate stability [144,145]. No tillage treatment results in enhancing the mean weight diameter of the aggregates (0.5 mm greater) compared with that under the PT system [146]. In another study, Zibilske and Bradford [147] reported that NT and reduced tillage (RT) showed significantly higher mean weight diameter in 0–5 and 10–15 cm soil depths than those under PT. The lower mean weight diameter and reduction in macro-aggregates under PT practice might be attributed to reduced soil aggregate stability and mechanical disruption of macroaggregates owing to continuous tillage operations. In micro-aggregates, the higher ratio of carbon/nitrogen (C/N) suggests that SOC accompanied with that fraction comprises less decay material and fundamentally consists of fungal hyphae and plant roots [87,144]. This shows accelerated changes in organic carbon induced by land use and management. In general, organic carbon encapsulated within the soil micro-aggregates is characterized as humified organic matter with a low C/N ratio [148]. Therefore, the adaptation of long-term conservation tillage practices, including NT and RT favors higher organic carbon and N concentrations, especially in upper soil profiles [149,150,151]. Working with different tillage operations, Chen et al. [152] reported that soils under CT practices contained 7.9 and 7.3% higher soil N concentration and SOC, respectively, than those under PT system in the surface layer (0–20 cm depth). Therefore, a long-term adaptation of NT and RT practices can play an essential role in maintaining and enhancing soil C content. Globally, 3.5% of Earth’s carbon reserves comprises soils, compared with 1% in biota and 1.7% in the atmosphere [124]. Under the CT system, incorporation of crop residues into the field maintains or increases SOC concentration by 4.9 × 1015 g C compared to 1.5 × 1015 g C under conventional tillage [13,153,154], which enhances aggregate stability [52,155,156,157]. Furthermore, as compared with SOC in macro-aggregates and labile fractions, it is reported that SOC in microaggregates has a longer turnover time and is usually more resistant to decomposition [60,158].

Under the ZT system, it is well known that soil microbial biomass C is mostly found to be higher under the CT system. Similarly, basal respiration (CO_2_ evolution) was often lower under the ZT system than under the CT, indicating lower specific respiration under the ZT system than under CT. Increased addition of biomass carbon and lower losses of labile C under ZT practice indicate the superior sequestration of C in the soil under the same tillage system. Conservation agricultural practices, including no-till agriculture, improve the efficacy of the agriculture system by improving water infiltration rates, reducing soil erosion losses, enhancing the stability of aggregates, promoting biological tillage, moderating soil environmental conditions (including temperatures), and reducing the weed populations. This system also reduces production cost and increases net profits, saves time, improves crop production through timely planting, decreases pest infestations through encouraging the soil biological communities, and lowers the emission of GHGs [159]. Thus, this system highly contributes to sequester more C and enhancing SOM content CT.

Degraded lands, also called agricultural marginal lands, are characterized as too steep or too shallow and too dry and too wet-lands and have been degraded by different environmental constraints, resulting in the decline in physio-chemical and biological properties of the soil. Principally, the marginal lands are degraded by accelerated soil erosion. However, a decline in soil structure, including soil compaction, salinization problems, nutrient and water imbalance, and invasion by obnoxious species, also contributes significantly to degradation processes. Therefore, the adaptation of NT practices helps to establish perennial land use and increases crop yield on degraded lands. Conversion to perennial land use contributes to sequestering soil C by 300–350 kg ha^−1^ yr^−1^ [34]. Under a traditional tillage system, soil disturbance reduces the SOC pool [160,161,162,163,164]; however, conversion of PT to NT increases the SOC pool, especially in upper soil layers (0–20 cm soil layer) [61,62]. Under NT system, there are several factors that defined the SOC sequestration rates. According to West and Post [36], the global SOC sequestration rates for conversion from PT to NT ranged between 400 and 600 kg ha^−1^ yr^−1^. However, according to Kimble et al. [33], these values ranged between 100 and 200 kg ha^−1^ yr^−1^ for the intensification of agricultural ecosystems. The adaptation of NT practice is more effective in SOC sequestration in lighter soils than soil with heavier texture [61,165,166]. Some other factors including the quality and quantity of plant biomass return to the field act as mulch and affect SOC sequestration rates [167,168]. Additionally, the antecedent SOC pool [169], landscape position, and slope gradient also affect the rate of SOC sequestration upon conversion to NT farming [170].

## 7. Conclusions and Future Perspectives

Careful management of natural resources is the most effective strategy to cope with the adverse effects of climate change on agriculture, which is a most sensitive sector relative to changing climatic conditions. Soil C sequestration, which involves transferring atmospheric CO_2_ into the soil, is a win-win approach that can deal with both climate adaptation and mitigation. The transformation of atmospheric CO_2_ into the soil primarily takes place through plant photosynthesis, and this process involves protecting the soil-based C pools from soil microbial populations that will release the C back to the atmosphere. The conversion of PT to NT system and the conversion of degraded and marginal lands to perennial land use are among the principal agricultural management practices of soil C sequestration. The no-till farming system sustains soil health and ecosystems and is considered as an effective strategy for restoring soil and sequestering the atmospheric C. Zero/no-tillage combined with the retention of crop residue into the field or use as mulch helps in sequestering a very significant portion of atmospheric CO_2_ and increasing water and nutrient use efficiency. Under a conservation agriculture system, crop rotation also shows promise for restoring soil and sequestering C because it contributes to increasing accumulation rates of SOC at various soil depths.

The need for drawdown solutions is progressively urgent and soil C sequestration through agricultural management practices warrants far better consideration from climate experts. Most, if not all, of the agricultural management practices that promote carbon sequestration also improve soil aggrege stability, water retention capacity, soil fertility status, and ensure food security. These noteworthy co-benefits should serve as incentives for augmented action. The significant discussion over the potential of soil CS will remain a bridge to the future. The protection dynamics along with soil C cycles are still not fully demonstrated in all locations on the globe, and variable patterns of land management make it difficult to predict the adoption of agricultural management practices that can sequester C. Nevertheless, a comprehensive understanding of soil C and the sequestration potential should not be a prerequisite for action. Recently, various research on different agricultural management practices has introduced numerous strategies to increase the sequestering of atmospheric C. Compared to a number of other atmospheric drawdown strategies, the adaptation of conservation tillage practices is relatively more effective and could be adapted in the near future. In this system, the risks are minimal while the known benefits of improving soil quality and sequestering C are numerous.
